# TCR repertoire shaping of naïve T cell subsets in human ontogeny

**DOI:** 10.3389/fimmu.2026.1738633

**Published:** 2026-03-25

**Authors:** Mikhail Yu. Myshkin, Sofya A. Leyn, Evgeny S. Egorov, Pavel V. Shelyakin, Dmitry B. Staroverov, Irina A. Shagina, Natalia E. Kan, Kseniia R. Lupyr, Ekaterina V. Barsova, Elena V. Dzyubinskaya, Yendry Ventura-Carmenate, Victor L. Tyutyunnik, Denis V. Rebrikov, Dmitriy M. Chudakov, Olga V. Britanova

**Affiliations:** 1 Shemyakin and Ovchinnikov Institute of Bioorganic Chemistry, Moscow, Russia; 2 Pirogov Russian National Research Medical University, Moscow, Russia; 3 Abu Dhabi Stem Cells Center, Abu Dhabi, United Arab Emirates; 4 Federal State Budget Institution (FSBI) «National Medical Research Center for Obstetrics, Gynecology and Perinatology Named After Academician V. I. Kulakov» Ministry of Health of the Russian Federation, Moscow, Russia; 5 Center for Molecular and Cellular Biology, Moscow, Russia; 6 Department of Immunology, Faculty of Biology, Lomonosov Moscow State University, Moscow, Russia

**Keywords:** Treg lymphocytes, CD4+, CDR2–MHC interactions, naïve CD8+, TCR repertoire, umbilical cord blood

## Abstract

T cell receptor (TCR) repertoire analysis provides crucial insight into the maturation of the adaptive immune system. In this study, we examined the diversity and structure of TCRβ repertoire in the sorted naïve CD4^+^, naïve CD8^+^ and naïve regulatory T cell (T_reg_) subsets from umbilical cord blood (UCB) at early gestation (24–29 weeks), term (38–39 weeks), and late-born neonates (40–42 weeks), as well as from peripheral blood of children, adults, and older individuals. UCB TCRβ repertoires were characterized by shorter CDR3 regions, increased repertoire convergence, and a higher abundance of public clonotypes, consistent with limited junctional diversity in early development. Our data suggest progressive maturation of the UCB TCRβ repertoire with noticeable changes by late gestation (~29 weeks). Notably, UCB-derived naïve T_reg_ cells displayed distinct TCRβ repertoire features compared with adult T_reg_ cells, indicating subset-specific differences in TCRβ repertoire shaping. Across age groups, we observed an age-dependent shift in TCRβ repertoire structure associated with changes in TRBV gene usage with a progressive decrease in the predicted binding strength of CDR2, particularly in repertoires of naïve T_reg_ and naïve CD8^+^; T cells. Together, these results provide insights into TCRβ repertoire formation during human ontogeny and highlight subset-specific trajectories during early life.

## Introduction

Development and maturation of the human adaptive immune system during gestation are of both fundamental and practical importance. Adaptive immune cells during human pregnancy undergo distinct maturation processes, establishing them as a specialized population with unique functions rather than simply immature versions of adult immune cells (1).

During human ontogenesis, hematopoiesis transitions sequentially through distinct anatomical sites: from the yolk sac to the fetal liver and subsequently to the fetal bone marrow. Generally, early-derived cells, including innate lymphoid cell precursors, provide innate-like protection, while later waves generate lymphoid progenitors (2). Unlike myeloid populations, the development of the T cell immune repertoire requires the formation and colonization of the thymus by lymphoid progenitors at approximately 7–8 weeks (3), after which it becomes fully functional with the export of T cells at 12–13 weeks of gestation (4). Critically, thymic colonization occurs in temporally distinct waves. An early wave generates innate-like lymphocytes, including tissue-resident γδ T cells and lymphoid tissue inducer cells essential for thymic medullary epithelial cell maturation. Subsequent waves produce conventional αβ T cells that form the adaptive immune repertoire. Importantly, each of the major fetal αβ T cell subpopulations (*e.g.*, regulatory T cells (T_regs_), conventional CD4^+^and CD8^+^, T cells) exhibits distinct phenotypic and functional properties compared with their adult counterparts.

Compared with adult naïve T cells, fetal naïve CD4^+^T cells show an increased intrinsic propensity to differentiate into T_regs_ (5), a property thought to be critical for establishing tolerance to maternal antigens and self-antigens (6). Fetal CD4^+^T helper cell differentiation is highly context-dependent: although neonatal vaccine studies reveal a predominant Th2 bias (7), fetal and neonatal T cells can mount robust Th1 responses in the setting of maternal infection or helminth antigen exposure (8), specialized tissue-resident PLZF^+^CD4^+^T cells in gut-associated lymphoid tissues produce IFN-γ, TNF-α, and IL-17 (9). In parallel, fetal-derived CD8^+^ T cells exhibit reduced granzyme B-mediated cytotoxicity compared with adult CD8^+^ T cells, yet display rapid, innate-like effector responses (10).

A key aspect of T cell immune development before birth is the shaping of the T cell receptor (TCR) repertoire, which establishes immune function at birth and influences susceptibility to infection, immune tolerance, and overall immune homeostasis throughout life (6, 11–13). TCR is formed through somatic recombination of V, J, and D (for the β chain) segments, accompanied by nucleotide additions and deletions at the junctions, resulting in the formation of the hypervariable third complementarity-determining region (CDR3). The quasi-stochastic process of V(D)J recombination generates a great diversity of TCRs, with the number of unique TCR beta chains in unfractionated human T cells estimated to be on the order of 10^8^–10^9^ (14) Across the postnatal human lifespan, TCR repertoire diversity increases in childhood, and partially contracts in old age, with well-documented changes in CDR3 length, junctional diversity, and clonal structure (13, 15–17).

During fetal development, TCR repertoires are characterized by shorter CDR3 sequences with minimal non-template nucleotide additions, creating a less diverse, more germline-encoded repertoire (13, 18). By late gestation, CDR3 length and repertoire diversity increase substantially (19). Following birth, TCR diversity gradually decreases with age (13, 17). Notably, older adults demonstrate a partial return to fetal-like repertoire features, including increased abundance of TCRs with shorter CDR3 regions and reduced non-template additions, potentially reflecting either selective survival of early-wave T cells generated during fetal life or preferential maintenance of innate-like TCR variants (16, 20, 21). However, direct comparative analyses of TCR repertoire formation across the human lifespan, especially within functional naïve CD4^+^, CD8^+^, and naïve T_reg_ subsets during fetal development, are still limited.

Here, we investigated the formation and maturation of TCRβ repertoires across human ontogeny, with a particular focus on naïve CD4^+^, naïve CD8^+^, and naïve T_reg_ cell subsets during fetal development and early life. Thus, this work provides a better understanding of fetal immune repertoire maturation at the TCR repertoire level.

## Results

**Figure 1:**
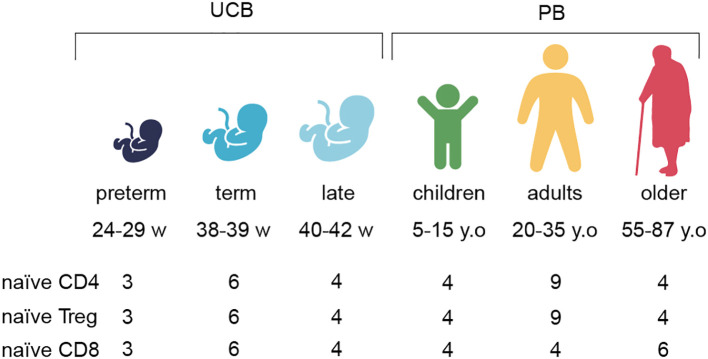
Overview of the included datasets. Naïve CD4 – naïve CD4^+^T lymphocytes, naïve T_reg_ – naïve regulatory CD4^+^T lymphocytes, naïve CD8 – naïve CD8^+^T lymphocytes. PB – peripheral blood. UCB samples were collected from 25 to 42 weeks of gestation (w.g.). PB donors were from 5 to 87 years old (y.o.). Icons from http://www.OnlineWebFonts.Com .

We analyzed TCR repertoires of naïve CD8^+^, naïve CD4^+^, and naïve T_reg_ lymphocytes in UCB from preterm (< 30 weeks), term, and late-born neonates. We compared these repertoires with corresponding naïve T cell subsets derived from peripheral blood samples of children, adults, and older adults (**Figure 1**).

We collected UCB samples ranging from 24 to 42 weeks of gestation (w.g.), isolated functional T cell subsets, and performed deep TCRβ repertoire profiling. Specifically, we sorted naïve ^-^7A;, naïve CD4^+^, and naïve T_reg_ populations from three distinct gestational stages: early preterm neonates (N = 3; 25–29 w.g.), term neonates (N = 6; 38–39 w.g.), and late-term neonates (N = 4; 40–42 w.g., **Figure 1**; **Supplementary Table 1**). To broaden the repertoire analysis, we included sequencing datasets from naïve CD8^+^, naïve T_reg_, and naïve CD4^+^ T cell subsets from children (**Supplementary Table 1**), adults (20–35 y.o.) and older adults (55–87 y.o.) previously published in (20, 22).

### An elevated frequency of non-functional TCR clonotypes characterizes UCB and older adult immune repertoires

**Figure 2:**
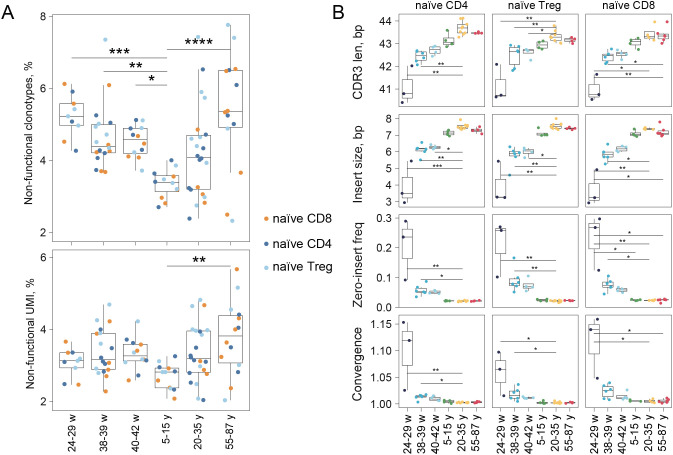
Analysis of basic features of TCRβ repertoires in UCB samples and in PB samples (children, adults, and older adults). **(A)** Proportion of non-functional clonotypes defined by unique TCR CDR3β nucleotide sequence **(top)** and proportion of non-functional TCR sequences by UMIs (bottom). Non-functional clonotypes are defined by the presence of out-of-frame and/or stop codons in CDR3β regions. **(B)** Characteristics (CDR3 length, insert size, zero-insert frequency) were calculated for datasets of functional clonotypes (**Supplementary Table 2**). Zero-insertion frequency shows the fraction of CDR3β containing zero-added nucleotides between V, D, and J-segments. Convergence is measured as the ratio of a number of different CDR3β nucleotide sequences to the number of different CDR3β amino acid sequences. For convergence calculation in each dataset, 3,000 most abundant clonotypes, defined as unique nucleotide sequences, were selected. Significance was calculated using the Kruskal-Wallis test with subsequent FDR correction and *post hoc* Dunn test. Only *p*-values < 0.05 are shown. ‘*’*p* ≤ 0.05, ‘**’*p* ≤ 0.01, ‘***’*p* ≤ 0.001, ‘****’*p* ≤ 0.0001.

We observed a developmental shift in the proportion of non-functional TCRβ clonotypes by the number of unique nucleotide sequences and by UMIs (unique molecular identifiers) between UCB and PB (peripheral blood) samples. The lowest fractions of non-functional clonotypes defined as unique TCR nucleotide sequences and non-functional TCR sequences counted by UMIs were detected in the immune repertoires of children (**Figure 2A**). The proportion of non-functional mRNA transcripts may reflect the efficiency of nonsense-mediated mRNA decay (NMD), which eliminates transcripts with premature stop codons (23). Similar increases in non-functional (out-of-frame) mRNAs in UCB TCRβ repertoires have been previously described (13). Notably, NMD efficiency can be modulated under cellular stress, as part of broader metabolic and transcriptional adaptation, potentially leading to its transient inhibition (24).

### Short CDR3s and high convergence characterize preterm TCRβ repertoires

Initially, we focused on analyzing the properties of the CDR3β repertoire, since it primarily mediates interaction with epitopes in the MHC context thus determining the functionality of the diverse TCR repertoire (25–27).

Quantitative analysis of CDR3β characteristics revealed a significant developmental progression in all T cell subsets. CDR3β length increased from preterm to term (38–39 w.g.) and continued to increase into the late−term period (40–42 w.g., **Figure 2B**). A corresponding increase in the frequency of random nucleotide insertions was observed during the same developmental window. These patterns are consistent with the developmental onset of terminal deoxynucleotidyl transferase (TdT) activity in the thymus. It was reported that TdT-positive thymocytes emerge after 20 w.g (28, 29). In mice lacking TdT, CDR3 length was found to be shorter than in wild-type mice (30), which supports the link between enzyme production onset and CDR3 lengthening.

The low TdT expression during early fetal development creates a distinctive molecular signature in preterm TCR repertoires. Clonotypes without inserted random nucleotides (zero-insertions, or zero-clonotypes) predominated in preterm samples among all T cell subsets (**Figure 2B**). In UCB repertoires, the proportion of zero-clonotypes declined sharply by the time of birth (38–39 w.g.). Additionally, the average number of identical amino acid CDR3β sequences encoded by different nucleotide sequences (*i.e.*, convergent clonotypes) was higher in preterm samples (**Figure 2B**), consistent with previously published data for sorted UCB CD8^+^ T cells and bulk UCB repertoire (13, 19). The zero-clonotype abundance strongly correlates with both high repertoire convergence and high clonotype publicity (21). In addition, overall repertoire diversity and clonality increased from the preterm stage toward term and late developmental stages (**Supplementary Figure 1**, **Supplementary Table 2**), as shown with the observed diversity and the normalized Shannon-Wiener index.

Our data indicate continued maturation of the repertoire by 40–42 w.g. with the most pronounced change between preterm (~29 weeks) and term periods.

### TCR repertoire similarity in unrelated UCB samples

**Figure 3:**
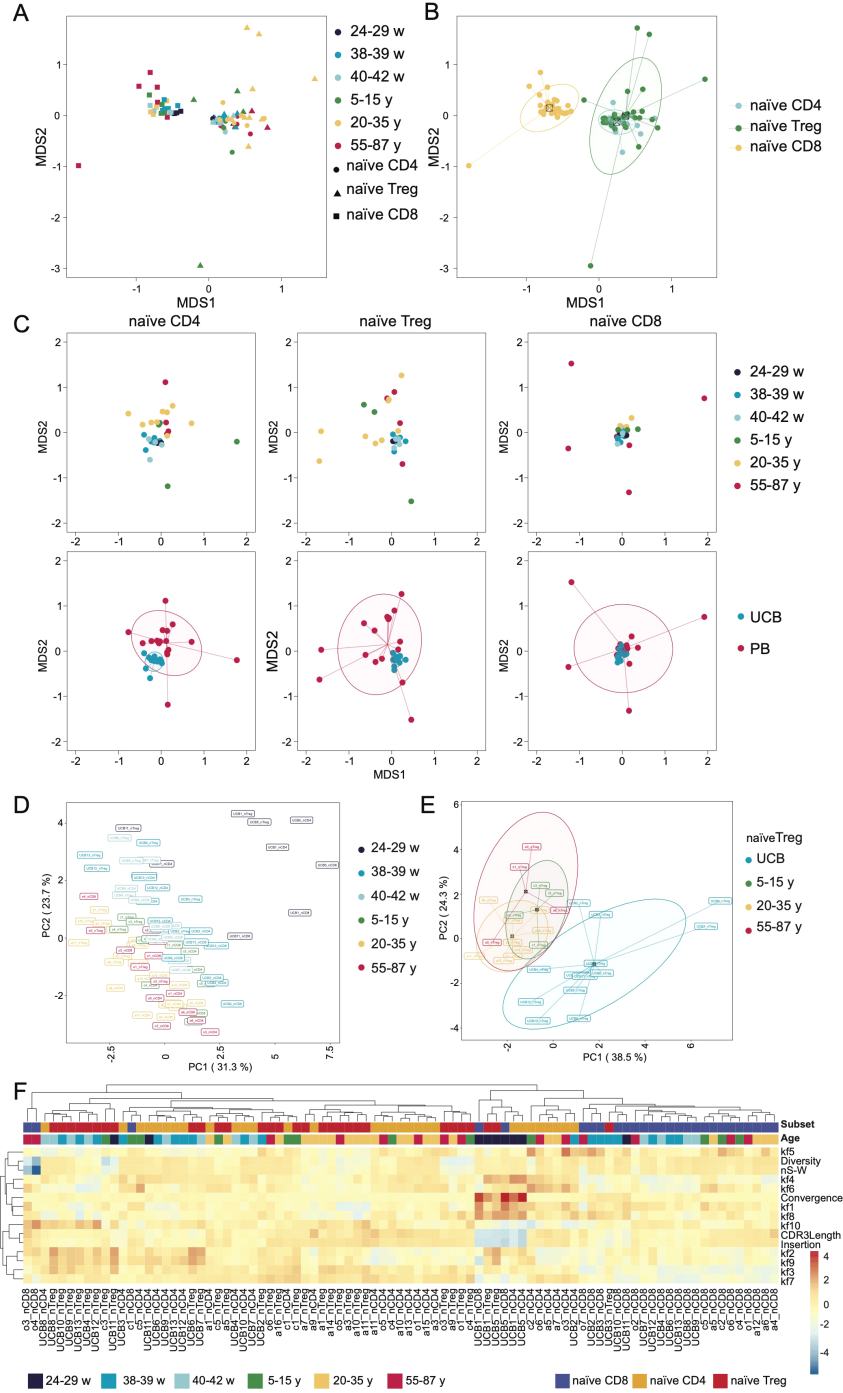
An overlap analysis of the TCRβ functional repertoires of naïve CD4^+^, naïve T_reg_, and naïve CD8^+^T cells sorted from UCB (preterm, term, late) and PB donors (children, adults, older adults). **(A)** MDS plots for all samples were constructed with the –log_10_(F2) metric as a distance measure between immune repertoires. Clonotypes were considered overlapping if they had the same V segment and the same CDR3 amino acid sequence. **(B)** Centroids and dispersions for cell subsets: CD4^+^samples (both naïve T_reg_ and naïve CD4^+^T cells) are clustered together and apart from naïve CD8^+^T cells. **(C)** MDS plots for separate functional subsets. The points in the upper panel are colored by age group. The lower panel shows the dispersions of UCB *vs*. PB samples. **(D)** Principal component analysis (PCA) of repertoire features, including Kidera factors 1–10 calculated for the five central amino acids within CDR3, convergence, CDR3 length, and repertoire diversity, with samples colored by age group. **(E)** PCA of the same feature set highlighting naïve T_reg_ repertoires, showing separation of UCB-derived naïve T_reg_ cells from postnatal age groups. **(F)** Hierarchical clustering heatmap of repertoire z-scored features across all samples.

To explore global repertoire similarity, we assessed the pairwise distance between all TCRβ repertoires using the sum of geometric means of frequencies for each pair of clonotypes from two different repertoires (F2 metric of VDJtools (31)) and visualized it using multidimensional scaling (MDS) (32). Notably, naïve CD8^+^ repertoires formed a distinct cross-donor cluster, while naïve CD4^+^ and naïve T_reg_ repertoires showed greater cluster overlap (**Figures 3A, B**). This pattern is consistent with known differences in MHC class I versus class II restriction during CD8^+^, and CD4^+^ T cell development, respectively, where the CDR3 repertoire is primarily shaped by peptide-MHC binding specificity rather than MHC binding alone (33). Indeed, numerous studies have demonstrated that peptide-MHC class I and class II complexes impose different interaction constraints on TCRs, resulting in different structural and sequence features in the CDR3β region (34–36).

Next, the overall repertoire similarity across age groups was analyzed separately for naïve CD8^+^, naïve CD4^+^, and naïve T_reg_ cell subsets. Consistent with the previous report on bulk repertoires (13), we observed a shared clonotype pattern within each of the cell subsets, characterized by pronounced linear sequence similarity. This close clustering on sequence distance was observed within UCB repertoires, whereas TCR repertoires from children, adults, and older adults had wider divergence within the group (**Figure 3C**). In MDS, the F2 metric captures the frequency-weighted overlap of shared clonotypes between samples and thus highlights the increased contribution of public clonotypes with relatively short CDR3s length in UCB repertoires. In summary, within cell subsets, UCB repertoires formed tight cross-donor clusters distinct from PB repertoires (**Figure 3C**).

Along with structural features of TCR repertoires such as convergence, CDR3β length, diversity, and similarity, we analyzed Kidera factors to characterize physicochemical properties of central amino acids with higher potential for epitope interaction (37). Kidera factors are ten features that describe protein–protein interface characteristics, including steric and chemical properties. We summarized Kidera factors 1–10 (kf) computed for the five central amino acids within CDR3 using hierarchical clustering and principal component analysis (PCA) (**Supplementary Table 2**, **Figures 3D–F**). Both approaches revealed that preterm repertoires formed a distinct cluster, separated from postnatal samples. This divergence is characterized by elevated kf8 (occurrence in alpha region), kf4 (hydrophobicity), and kf1 (alpha-helix/bend-structure preference), reduced kf9 (pK-C), increased convergence, and shorter CDR3 length. Notably, the UCB11 sample (29 w.g.) showed partial separation from other preterm samples at 24 and 25 w.g. (**Supplementary Table 1**). This may reflect a transition stage toward increased TdT contribution. Taking these differences into account, we additionally calculated the frequency of amino acid residues within the central region of CDR3 (**Supplementary Figures 2****–****4**). In the preterm repertoire, polar residues such as Ser, Thr, and Tyr, which are capable of forming hydrogen bonds, were enriched. In contrast to PB repertoires, hydrophobic residues Pro and Val were underrepresented in UCB repertoires. Notably, the charged and polar residue His was highly abundant in UCB T_reg_ cells, while Gly was relatively enriched in PB T_reg_ cells (**Supplementary Figure 3**). To reduce the impact of CDR3 length and correlated V/J-linked biases, we downsampled repertoires to adjust the CDR3 length distributions across all age groups and recomputed residue frequencies. The observed trends remained consistent.

In general, UCB naïve T_reg_ repertoires noticeably diverged from T_reg_ repertoires of other age groups (**Figure 3E**). Although the size of the preterm group (N = 3) and overall UCB group (N = 13) was rather limited, we speculate that this may indirectly reflect unique features of selection during early gestation and/or subsequent migration to specialized tissue niches after birth (38). Consistent with this notion, UCB naïve T_reg_ cells have been shown to demonstrate distinct functional properties with some studies reporting enhanced suppressive capacity (38) while others reports hyposuppression and epigenetic instability (39).

### Developmental changes in V-segment usage during TCR repertoire maturation

The intrinsic V-usage bias has recently been demonstrated in 201 Chinese newborn samples, providing evidence that MHC polymorphisms influence the usage of specific TRBV genes (40). This work highlights that germline-encoded contacts influence TCR–MHC compatibility early in ontogeny prior to antigen-specific exposure, supporting the hypothesis that thymic selection may already be influenced by inherited MHC–TCR V gene pairings.

**Figure 4:**
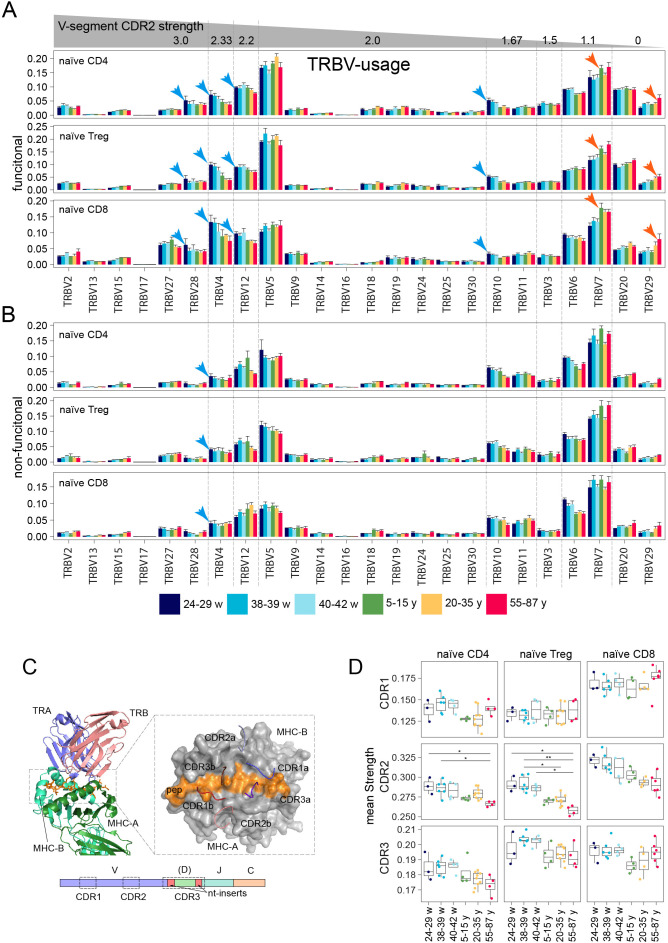
Analysis of V-gene usage in the TCRβ functional **(A)** and non-functional **(B)** repertoire of naïve CD8^+^, naïve CD4^+^, and naïve T_reg_ cells sorted from UCB (preterm, term, late) and PB (children, adults, older adults). V-segment families are sorted by their CDR2 binding strength. Blue arrows indicate TRBV segments more highly expressed in UCB samples, red arrows indicate those with higher expression in adult samples. **(C)** Structure of TCR-pMHC-II complex (43). Classical TCR-MHC orientation: CDR3 of TCRα and TCRβ contact the peptide, while CDR1 and CDR2 contact the MHC molecule; CDR1 and CDR2 of TCRα bind MHC-B, and CDR1 and CDR2 of TCRβ bind MHC-A (44). **(D)** Analysis of strength in the TCRβ repertoire of naïve T cell subsets. Average values of the binding strength of amino acid residues included in the complementarity-determining regions (CDR1, CDR2, CDR3) according to the IMGT database in repertoires of naïve CD4^+^, naïve T_reg_, naïve CD8^+^T cells in UCB (preterm, term, late) and children, adults, older adults. For the third row, only 5 central residues of CDR3 were used. Significance was calculated using the Kruskal-Wallis test with subsequent FDR correction and *post-hoc* Dunn test. Only *p*-values < 0.05 are shown. ‘*’*p* ≤ 0.05, ‘**’.

Our work complements these findings by analyzing TRBV usage profiles in repertoires of naïve CD4^+^, naïve T_reg_, and naïve CD8^+^, T cells across age groups. Certain TRBV segments were preferentially represented in either UCB or PB naïve T cell repertoires (**Figure 4**). For instance, TRBV28 was preferentially used in the functional repertoire only in preterm TCR repertoires in all three subsets. TRBV4, TRBV10, and TRBV12 were abundantly represented in UCB repertoires, while the usage frequency of TRBV7 and TRBV29 was higher in the adult or older adult repertoires (**Figures 4A, B**).

We hypothesized that differences in TRBV gene usage between UCB and adult samples could influence the overall properties of the TCR repertoire. To assess this, we calculated the predicted binding strength of the germline-encoded CDR1β and CDR2β regions, which typically contribute to TCR interactions with MHC molecules (27). In contrast, the hypervariable CDR3β region primarily contributes to antigen recognition (20, 25), as schematically illustrated in **Figure 4C**. Amino acid residues contribute unequally to TCR-MHC contacts, which is supported by a theoretical model from Košmrlj and Chakraborty of thymic selection (41). The predicted binding strength is determined by the average number of strongly binding amino acid residues in the central part of CDRs (L, F, I, M, V, W, C, Y), which are expected to promote stronger van der Waals and hydrophobic contacts between amino acid residues. Thus, higher “strength” values indicate a greater potential for strong CDR-MHC interactions. For the central five amino acid residues within CDR3, we also computed the predicted binding strength. This parameter has been previously shown to distinguish TCR repertoires of T_reg_ and conventional CD4^+^T cells in mice, consistent with the higher TCR affinity range allowed during thymic selection of T_reg_ cells (32, 42).

In contrast to CDR1β, we observed a trend of decreasing CDR2 binding strength with age in naïve CD8^+^, naïve CD4^+^ T cells and was most prominent in the naïve T_reg_ subsets when comparing UCB and PB samples (**Figure 4D**). Notably, TRBV4, which encodes a CDR2 with high predicted binding strength, was overrepresented in functional UCB repertoires following selection, whereas no comparable enrichment was observed in non-functional rearrangements. This suggests that clonotypes utilizing this TRBV segment might be constrained by selection rather than solely stochastic recombination events. Conversely, usage of the TRBV7 segment, which encodes a CDR2 region with low binding strength, tends to increase with age, particularly in naïve CD4^+^ and naïve T_reg_ T cells of older adults.

Depletion analysis of clonotypes containing particular TRBV sets, such as (TRBV4, TRBV7) or (TRBV4, TRBV7, TRBV29) showed progressively weakened age-dependent changes in CDR2β binding strength in naïve CD4^+^, naïve T_reg_, and naïve CD8^+^; T cell repertoires (**Supplementary Figures 5A–D**). Age-dependent differences in CDR3β strength followed the same trend between UCB and PB age groups (**Supplementary Figure 5F**), with UCB repertoires exhibiting higher CDR3β binding strength compared to children. In line with the previous report (20), the average CDR3β strength in the repertoire of naïve CD4^+^ T cells tends to decrease with age, whereas the opposite tendency is observed in CD8^+ ^T cells (**Figure 4D**). Notably, CDR3 strength in the CD8^+^, repertoire drops sharply from UCB to children, and then increases progressively, rendering samples from older individuals more UCB-like in this respect (**Supplementary Figure 5F**).

Taken together, these repertoire-level metrics in the repertoire of naïve T cells are consistent with age-dependent dynamics in TCR repertoire in which germline-encoded CDR2β features could contribute more prominently during early T cell development. Concomitant with age, CDR3 contributes increasingly more to repertoire shaping, particularly in naïve CD8^+^, and CD4^+^ T cell subsets.

### Enrichment of virus-annotated TCRs in UCB repertoire reflects public clonotypes with high generation probability

To assess the fraction of viral-annotated TCR matches in UCB and adult repertoires, we extracted TCRβ clonotypes from VDJdb (45) with known specificity for CMV, EBV, SARS-CoV-2, and HIV-1 peptides presented by HLA-I. High-confidence virus-specific annotations are substantially more abundant for HLA-I (CD8^+^) than for HLA-II (CD4^+^), and therefore, our analysis was focused on only CD8^+^-specificities, whereas the number of HLA class II–restricted matches was insufficient for robust comparative analysis.

**Figure 5:**
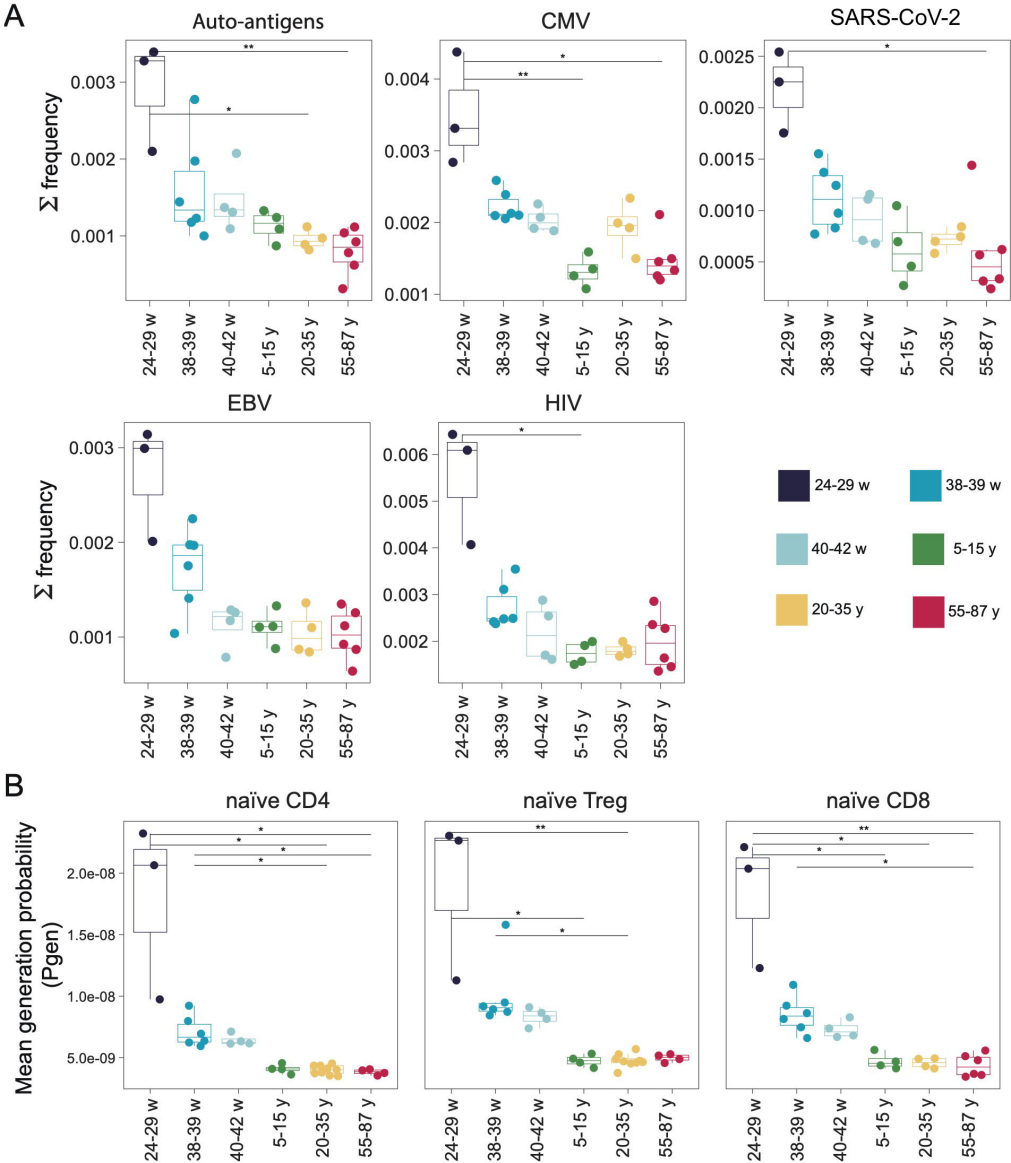
**(A)** Frequency of autoantigens (H) sapiens, CMV, SARS-CoV-2, EBV, and HIV-specific TCR clonotypes within repertoires of naïve CD8^+^T cells across different age groups. All repertoires were downsampled to 5000 random UMIs per sample to control sequencing depth. Clonotypes were considered positive if they shared the same V segment and a similar CDR3β amino acid sequence with at most 1 mismatch. CMV, EBV, SARS-CoV-2, HIV-specific clonotypes and autoantigen-specific clonotypes were extracted from VDJdb with a confidence score ≥ 1 and HLA class I annotations. **(B)** Mean generation probability (Pgen) of the intersecting clonotype sets, calculated using the OLGA framework. Significance was calculated using the Kruskal-Wallis test with subsequent FDR correction and *post hoc* Dunn test. Only *p*-values < 0.05 are shown. ‘*’*p* ≤ 0.05, ‘**’*p* ≤ 0.01.

From VDJdb we selected 872, 546, 808, and 329 clonotypes annotated as CMV-, EBV-, HIV-, and SARS-CoV-2-specific, respectively, as well as 419 autoantigen-specific clonotypes, all with confidence scores ≥ 1. We observed that the preterm group tended to have a higher cumulative frequency of virus-specific clonotypes and autoantigens (**Figure 5A**). Notably, this effect could be linked to the shorter CDR3 variants in preterm repertoires, which are more likely to be public and yield matches in VDJdb. Virus-specific clonotypes identified via VDJdb consistently had shortened CDR3 regions compared to the overall repertoire distribution, creating an increased probability of sequence intersection between preterm samples and known viral specificities (**Supplementary Figure 6**). As expected, intersecting clonotypes demonstrated significantly higher generation probabilities (P_gen_) calculated using OLGA software (46) compared to the average repertoire generation probabilities (**Figure 5B**).

The presence of such clonotypes in UCB repertoires can be considered a comparative feature of repertoire structure, reflecting intrinsic properties such as publicity, higher generation probability, and structural similarity to previously described antigen-specific TCRs. In this context, virus-annotated TCRs serve as reference sequences for assessing repertoire overlap and organization rather than as definitive indicators of functional antigen recognition.

## Discussion

In this study, we analyzed the repertoires of sorted naïve T cell subsets from UCB and compared them with PB T cell repertoires of children, adults, and older adults published previously (20, 22). Our focus was to examine the contribution of these early repertoires to the adult TCR landscape and the mechanisms underlying repertoire maturation.

As expected, CDR3β regions were shorter in preterm neonates, consistent with fewer insertions and a higher proportion of zero-insert clonotypes. This pattern likely reflects limited TdT activity in early gestation (28, 29) and preferential selection for shorter CDR3β loops in MHC-restricted TCRs during thymic selection (47). As previously shown, decreased TdT expression limits junctional diversity both in TCRs and BCRs, and TdT expression continues to increase not only in fetal but also more strongly in postnatal development within lymphoid cells (30, 48). Moreover, positive selection has been proposed to favor promiscuous, cross-reactive “public” TCRβ sequences in the human thymus, allowing the repertoire to respond to a wide range of epitopes in early life (49).

Notably, preterm TCRβ repertoires have been reported to contain a significant proportion of clonotypes that have undergone rearrangement without the D-segment, in contrast to the repertoires of infants and adults. This suggests that a different recombination scenario may occur during the early fetal period and later (50).

These features result in greater repertoire convergence and publicity. In line with this, it was shown that the TCR repertoire of newborns appeared closest to the germline and was enriched in public clonotypes that were shared across individuals (13).

We observed elevated frequencies of non-functional TCRβ transcripts in UCB repertoires, which may reflect developmental regulation of nonsense-mediated mRNA decay (NMD). It is well established that cells adapt to physiological stress by modifying gene expression and metabolism, and suppression of NMD is one such adaptive mechanism. Notably, hypoxia is a physiological stress factor common to both fetal development and aging (51, 52). The question remains as to why naïve T cell subsets are likely susceptible to this process. In support of this, studies in *C. elegans* have demonstrated an age-associated decline in NMD activity (53). This suggests that the increased abundance of non-functional TCR transcripts in UCB and older adult repertoires may be at least partly driven by hypoxia-induced downregulation of NMD during these stages of life.

Moreover, recent studies indicate that newborns experience significantly lower capillary oxygen saturation during the first minute after birth, with levels normalizing within 10 minutes postpartum, a transition that occurs more slowly in preterm infants (54). These data likely reflect transient perinatal hypoxia associated with delivery, rather than sustained intrauterine hypoxemia during non-pathological fetal development. Thus, the putative hypoxia-related modulation of NMD may arise not from chronic low oxygen levels *in utero*, but from acute physiological stress occurring around birth.

Analysis of TCRβ CDR features suggests a contribution of germline-encoded CDR2β to the repertoire shaping of naïve CD4^+^, naïve T_reg_, and naïve CD8^+^ T cells during ontogenesis, although this impact, assessed by potential binding strength, gradually diminishes throughout the lifespan. Collectively, our observations indirectly support the model that early thymic selection can rely more on germline-biased interactions of TCR-pMHC, while highly diverse CDR3β regions become more influential after birth.

Based on the clonotype sharing and MDS analysis, we examined how repertoire organization relates to functional identity and developmental stage. Similarity analysis of clonotypes revealed that TCR repertoires clustered more strongly by functional subset (naïve CD4^+^, naïve T_reg_, naïve CD8^+^ T cells) than by donor age, highlighting that functional identity exerts a dominant influence on repertoire structure, even across developmental stages.

To explore age-dependent changes in more detail, we assessed overall similarity between the CDR3 repertoires, focusing on the impact of physicochemical properties of the five central amino acid residues within the region. PCA and heatmap analyses of repertoire features across different age groups revealed that UCB naïve T_regs_ clustered separately from peripheral blood samples, indicating a distinct early-life repertoire feature profile. The TCR repertoires in preterm infants display increased frequencies of polar amino acid residues in the central region of CDR3 (e.g., His, Tyr for naïve T_reg_, Ser, Thr, Tyr for naïve CD8^+^ T cells, Tyr, Ser for naïve CD4^+ ^T cells). This may increase the availability of hydrogen-bonding contacts at the binding interface and, hypothetically, may contribute to cross-reactivity and a broader range of peptide-contact geometries early in life (49).

We next investigated the antigen recognition potential of these developmentally distinct repertoires. Using CMV, HIV, SARS-CoV-2, and EBV-specific CD8^+^ TCRs from VDJdb, we observed an enrichment of these specificities within the naïve CD8^+^ TCR repertoires in preterm infants compared with older groups. The observed enrichment of virus-annotated clonotypes in UCB repertoires should not be interpreted as direct evidence of functional antiviral immunity. Recent large-scale benchmarking studies have demonstrated that a substantial fraction of TCRs annotated as virus-specific in public databases cannot be functionally validated under experimental conditions (55). This enrichment likely reflects the higher abundance of public, germline-like TCRs in early life, with a higher probability of generation, which requires zero to minimal involvement of the TdT enzyme in the VDJ rearrangement mechanism.

Similar to our predicted virus-specific functionality of preterm clonotypes, TdT-knockout mice have been shown to exhibit highly diverse responses at the repertoire level to various viral epitopes. However, these responses were characterized by an altered hierarchy of epitope dominance, suggesting that TdT-mediated junctional diversity was not required for response breadth but played a key role in shaping epitope immunodominance (56). A recent study showed that, in contrast to adult CD8+ T cells, neonatal T cells can undergo rapid clonal expansion upon activation but preferentially differentiate into short-lived effector populations with elevated MAPK and mTOR signaling pathways (57).

Our findings may be of interest not only from a fundamental perspective but also to clinicians, as UCB-derived T_regs_ are under active clinical development as cellular therapies across multiple immune−mediated diseases. Key applications are currently in the treatment of graft−versus−host disease (GVHD (58),, bone marrow failure syndromes (59), and hyperinflammatory syndromes such as COVID-19-associated Acute Respiratory Distress Syndrome (ARDS (60). Previous studies have demonstrated that UCB T_regs_ exhibit a distinct and more homogeneous transcriptomic and functional profile compared to adult PB T_regs_. Specifically, UCB T_regs_ maintain elevated expression of key regulatory molecules such as FOXP3, CTLA-4, CD39, and GARP, and consistently demonstrate superior suppressive capacity *in vitro* (38).

The main limitations of this study are the low number of preterm samples and the absence of HLA genotyping data. Some of our conclusions rely on differences in TRBV gene usage, which can be influenced by HLA background. Although thymic selection occurs in an HLA-dependent context, recent large-scale analyses have demonstrated extensive TCR cross-reactivity across multiple HLA alleles. In particular, TCR-based HLA similarity networks revealed that HLA alleles can be grouped into functional clusters sharing overlapping TCR repertoires (35). This might explain the relatively modest HLA-associated influences on TCR repertoire structure compared with the dominant contributions of V(D)J recombination. Consequently, the pronounced variability observed among adult repertoires could be shaped to a much greater extent by the individual history of antigen encounters than, for instance, by HLA-associated genetic factors.

In summary, we profiled TCRβ repertoires of sorted naïve CD4^+^, naïve CD8^+^, and naïve T_reg_ subsets across human ontogeny, including multiple gestational stages of UCB. Our observations are consistent with a model in which germline-biased TCR-pMHC interactions contribute significantly in early life. Across ontogeny, repertoire organization remains dominated by functional subset identity, while age primarily influences diversity and compositional repertoire features. Our data imply that developmental differences are already evident by late gestation (~29 weeks).

## Materials and methods

### Sample collection and cell sorting

UCB for extremely preterm (25–29 w.g.), term (38–39 w.g.), and late (40–42 w.g.) newborns were obtained in collaboration with the FSBI «National Medical Research Center for Obstetrics, Gynecology and Perinatology named after V. I. Kulakov». PB samples of 7–9 ml were collected from children (5–16 y.o.), adults (20–35 y.o.) and older adults (65–83 y.o.). The study protocol was reviewed and approved by the Ethics Committee of Kulakov National Medical Research Center for Obstetrics, Gynecology and Perinatology (Approval Reference: No. 2017/48).

For UCB, cell sorting was performed using monoclonal antibodies against CD4-FITC (OKT4), CD3-eFluor450 (Ucht1), CD25-APC (BC96). The following cell populations were collected: naïve T_reg_ (CD3^+^CD4^+^CD25^+^), naïve CD4^+^(CD3^+^CD4^+^), naïve CD8^+^(CD3^+^CD4^-^) T cells. Additionally, two naïve CD4^+^samples (UCB12, UCB13) from the term group and children were sorted as mature naïve CD4^+^(CD3^+^CD4^+^CD25^-^CD45RA^+^CD27^+^CD31^-^), T_reg_ (CD3^+^CD4^+^CD25^+^CD45RA^+^CD27^+^), CD8^+^(CD3^+^CD4^-^CD45RA^+^CD27^+^) with mix: CD4-APC-Cy7 (13B8.2), CD25-PE (BC96), CD31-AlexaFluor647 (WM59), CD27-eFluor450 (O323), and CD45RA-FITC (JS-83) antibodies.

Details on the sorting and treatment of samples from adults, and older adults were published (22). PBMC samples were sorted with mix described above using the following parameters: naïve CD4^+^(CD4^+^CD45RA^+^CD27^+^CD31^-^CD25^-^), a cell population enriched in naïve T_regs_ (CD4^+^CD45RA^+^D25^+^), and naïve CD8^+^T lymphocytes (CD8^+^CD45RA^+^CD27^+^).

Isolation of the mononuclear fraction of cells was performed using centrifugation in a Ficoll gradient. Cell sorting of T cell populations was performed with the BD FACS Aria III system. Cells were sorted directly into RLT lysis buffer (Qiagen) at a rate of 350 µl of buffer per 100,000 sorted T cells and kept at -70C.

### Preparation of cDNA libraries of TCR repertoires

For the extraction of total RNA from sorted cell populations, the RNeasy Micro kit (Qiagen) was used according to the manufacturer’s protocol. cDNA synthesis and amplification with the introduction of molecular barcodes (UMIs) were performed using the protocol 5’RACE TCRαβ Kit Human (LLC MiLaboratory). Sequencing was performed using Illumina HiSeq 2500 and Illumina NextSeq 550 platforms with paired 150 + 150 reads.

### Raw data preprocessing

The MiGEC (version 1.2.7) and MiXCR (version 3.0.13 (61–63), were used for data preprocessing. UMIs were extracted from raw sequencing reads using MiGEC software with a threshold of a minimum 2 reads per UMI for all samples (**Supplementary Table 1**). Reads sharing identical UMIs were grouped into molecular groups (MIGs); MIGs containing only one read were excluded from further analysis. Next, consensus sequences derived from each MIG were aligned to reference germline sequences of V, D, and J TCRB gene segments and assembled into clonotypes using MiXCR.

### Post-analysis

Averaged physicochemical parameters of the hypervariable CDR3β region amino acid sequences, diversity metrics of TCR repertoires, and their intersections were calculated using the repseq Python library (https://github.com/mmjmike/repseq/). For the “convergence” metric, we used 3000 most abundant clonotypes from each sample. For observed diversity and normalized Shannon–Wiener index we performed downsampling to 4500 randomly chosen UMIs. The R-language was used for data manipulation, plotting, and statistics.

For pairwise sample intersections, the F2 metric was used – the sum of geometric means of frequencies for each similar pair of clonotypes from two different repertoires. Clonotypes were considered similar if they shared the same V segment and the same CDR3β amino acid sequence. We plotted their relative distances in 2D using multidimensional scaling (MDS) and *–log_10_(F2)* metric as the distance measure.

Naïve CD4^+^and naïve T_reg_ repertoires were downsampled to 15–000 functional UMIs for intersection with effector repertoires while naïve CD8 repertoires were downsampled to 5000 functional UMIs for intersection with antigen-specific clonotypes from VDJdb.

Lists of public human TCRβ clonotypes specific to CMV, EBV, SARS-CoV-2 and HIV-1 peptides in complex with MHC-I were extracted from VDJdb (version from June 2023) and filtered for having a confidence score at least 1.

PCA was performed on the following features of the repertoires: convergence, observed diversity, normalized Shannon–Wiener index, mean insert size, mean CDR3nt length, and mean Kidera factors 1–10 – all calculated as described above. Each of the features was z-score standardized among all the samples (“scale” function in R) prior to running the PCA (“pca” function in R).

## Data Availability

Sequencing data that support the findings of this study are available in the NCBI Sequence Read Archive under BioProject accession PRJNA1431179 (https://www.ncbi.nlm.nih.gov/bioproject/PRJNA1431179). Preprocessed repertoires and metadata are available in Figshare: https://doi.org/10.6084/m9.figshare.31452001.

## Glossary

UCB: umbilical cord blood
PB: peripheral blood
PBMC: peripheral blood mononuclear cells
UMI: unique molecular identifier
NMD: nonsense-mediated mRNA decay
TCR: T-cell receptor
TCRβ: T-cell receptor chain Beta
TRA: T-cell receptor chain Alpha
TdT: terminal deoxynucleotidyl transferase
CDR: complementarity-determining region
CMV: cytomegalovirus
MDS: multidimensional scaling
