# Supplementary material for: TCR repertoire shaping of naïve T cell subsets in human ontogeny
Source: Front Immunol. 2026 Mar 25;17:1738633. doi: 10.3389/fimmu.2026.1738633 (PMC13057321; doi:10.3389/fimmu.2026.1738633)
Supplement: Supplementary file 1 [file Supplementaryfile1.docx]

***Supplementary Material***

# **Supplementary Data**

**Table S1.**

Sequencing data on UCB and children, adult, older adult samples of naïve CD4+, CD8+ and Treg cell subsets. Adult and older adult samples were previously published [1, 2].

| **Sample_id** | **Age** | **Experimental group** | **Cell subset** | **Chain** | **UMI groups** | **Functional singletons** | **Clonotypes** | **Functional clonotypes** |
| --- | --- | --- | --- | --- | --- | --- | --- | --- |
| UCB4_nCD4 | 42w | late | naïve CD4 | TRB | 24297 | 8453 | 15004 | 14235 |
| UCB7_nCD4 | 40.5w | late | naïve CD4 | TRB | 71461 | 24999 | 44002 | 41850 |
| UCB8_nCD4 | 40w | late | naïve CD4 | TRB | 95685 | 25730 | 51977 | 49528 |
| UCB9_nCD4 | 41.5w | late | naïve CD4 | TRB | 213888 | 57166 | 115560 | 110597 |
| UCB4_nCD8 | 42w | late | naïve CD8 | TRB | 27886 | 8248 | 15945 | 15199 |
| UCB7_nCD8 | 40.5w | late | naïve CD8 | TRB | 19808 | 13016 | 16440 | 15764 |
| UCB8_nCD8 | 40w | late | naïve CD8 | TRB | 53974 | 21884 | 35689 | 34094 |
| UCB9_nCD8 | 41.5w | late | naïve CD8 | TRB | 198653 | 54031 | 108301 | 103879 |
| UCB4_nTreg | 42w | late | naïve Treg | TRB | 23297 | 8567 | 14612 | 13896 |
| UCB7_nTreg | 40.5w | late | naïve Treg | TRB | 26997 | 14718 | 20517 | 19468 |
| UCB8_nTreg | 40w | late | naïve Treg | TRB | 33631 | 18858 | 25709 | 24753 |
| UCB9_nTreg | 41.5w | late | naïve Treg | TRB | 35354 | 16312 | 24602 | 23562 |
| UCB1_nCD4 | 25w | preterm | naïve CD4 | TRB | 100467 | 25965 | 52696 | 50019 |
| UCB5_nCD4 | 24w | preterm | naïve CD4 | TRB | 104347 | 21731 | 49366 | 46447 |
| UCB11_nCD4 | 29w | preterm | naïve CD4 | TRB | 143556 | 28126 | 64477 | 61724 |
| UCB1_nCD8 | 25w | preterm | naïve CD8 | TRB | 76354 | 23539 | 43330 | 40913 |
| UCB5_nCD8 | 24w | preterm | naïve CD8 | TRB | 106163 | 20983 | 48765 | 45777 |
| UCB11_nCD8 | 29w | preterm | naïve CD8 | TRB | 148419 | 29398 | 69412 | 66274 |
| UCB1_nTreg | 25w | preterm | naïve Treg | TRB | 84044 | 19180 | 40836 | 38701 |
| UCB5_nTreg | 24w | preterm | naïve Treg | TRB | 56801 | 13662 | 28999 | 27426 |
| UCB11_nTreg | 29w | preterm | naïve Treg | TRB | 16744 | 3841 | 8068 | 7667 |
| UCB2_nCD4 | 38w | term | naïve CD4 | TRB | 139476 | 44542 | 81956 | 78511 |
| UCB3_nCD4 | 38.5w | term | naïve CD4 | TRB | 55515 | 25164 | 38736 | 36701 |
| UCB6_nCD4 | 38.5w | term | naïve CD4 | TRB | 34149 | 14572 | 23056 | 22069 |
| UCB10_nCD4 | 38w | term | naïve CD4 | TRB | 262503 | 54212 | 125688 | 119344 |
| UCB12_nCD4 | 38.5w | term | naïve CD4 | TRB | 34663 | 22218 | 28150 | 27005 |
| UCB13_nCD4 | 38.5w | term | naïve CD4 | TRB | 40266 | 25049 | 32131 | 30931 |
| UCB2_nCD8 | 38w | term | naïve CD8 | TRB | 146591 | 41504 | 81317 | 77851 |
| UCB3_nCD8 | 38.5w | term | naïve CD8 | TRB | 85420 | 31675 | 54212 | 50909 |
| UCB6_nCD8 | 38.5w | term | naïve CD8 | TRB | 72627 | 20085 | 39762 | 38299 |
| UCB10_nCD8 | 38w | term | naïve CD8 | TRB | 145678 | 48497 | 87324 | 83632 |
| UCB12_nCD8 | 38.5w | term | naïve CD8 | TRB | 24244 | 16504 | 20368 | 19465 |
| UCB13_nCD8 | 38.5w | term | naïve CD8 | TRB | 55050 | 32843 | 42841 | 41255 |
| UCB2_nTreg | 38w | term | naïve Treg | TRB | 107687 | 27786 | 56686 | 54011 |
| UCB3_nTreg | 38.5w | term | naïve Treg | TRB | 37284 | 10146 | 20308 | 18814 |
| UCB6_nTreg | 38.5w | term | naïve Treg | TRB | 16512 | 9396 | 12691 | 12139 |
| UCB10_nTreg | 38w | term | naïve Treg | TRB | 129399 | 26484 | 60718 | 57720 |
| UCB12_nTreg | 38.5w | term | naïve Treg | TRB | 7082 | 4530 | 5778 | 5488 |
| UCB13_nTreg | 38.5w | term | naïve Treg | TRB | 10381 | 6264 | 8158 | 7790 |
| c1_nCD4 | 9 | children | naïve CD4 | TRB | 45792 | 25394 | 34691 | 33429 |
| c2_nCD4 | 12 | children | naïve CD4 | TRB | 6084 | 3481 | 4667 | 4508 |
| c4_nCD4 | 7 | children | naïve CD4 | TRB | 14155 | 6818 | 9912 | 9515 |
| c5_nCD4 | 10 | children | naïve CD4 | TRB | 32840 | 21374 | 26673 | 25952 |
| c1_nCD8 | 9 | children | naïve CD8 | TRB | 68155 | 32249 | 47503 | 45987 |
| c2_nCD8 | 12 | children | naïve CD8 | TRB | 67427 | 30467 | 45437 | 44160 |
| c4_nCD8 | 7 | children | naïve CD8 | TRB | 53145 | 26478 | 37919 | 36571 |
| c5_nCD8 | 10 | children | naïve CD8 | TRB | 44806 | 27783 | 35516 | 34463 |
| c1_nTreg | 9 | children | naïve Treg | TRB | 16012 | 8928 | 12167 | 11744 |
| c3_nTreg | 8 | children | naïve Treg | TRB | 4756 | 2265 | 3307 | 3201 |
| c4_nTreg | 7 | children | naïve Treg | TRB | 6622 | 3409 | 4819 | 4633 |
| c5_nTreg | 10 | children | naïve Treg | TRB | 10177 | 5724 | 7674 | 7415 |
| a1_nCD4 | 29 | young | naïve CD4 | TRB | 39766 | 17282 | 26520 | 25444 |
| a3_nCD4 | 32 | young | naïve CD4 | TRB | 64515 | 34089 | 47630 | 45716 |
| a5_nCD4 | 25 | young | naïve CD4 | TRB | 118837 | 21910 | 52283 | 48869 |
| a7_nCD4 | 26 | young | naïve CD4 | TRB | 22940 | 10071 | 15395 | 14760 |
| a9_nCD4 | 31 | young | naïve CD4 | TRB | 36490 | 16874 | 24536 | 23743 |
| a10_nCD4 | 26 | young | naïve CD4 | TRB | 64662 | 26583 | 42295 | 40299 |
| a11_nCD4 | 24 | young | naïve CD4 | TRB | 43923 | 16492 | 27477 | 26285 |
| a13_nCD4 | 30 | young | naïve CD4 | TRB | 43351 | 25054 | 33295 | 32233 |
| a15_nCD4 | 28 | young | naïve CD4 | TRB | 39413 | 23876 | 30937 | 30199 |
| a4_nCD8 | 45 | young | naïve CD8 | TRB | 51497 | 39357 | 45378 | 43989 |
| a5_nCD8 | 25 | young | naïve CD8 | TRB | 16879 | 12881 | 15111 | 14377 |
| a6_nCD8 | 30 | young | naïve CD8 | TRB | 21232 | 17800 | 19712 | 19070 |
| a12_nCD8 | 30 | young | naïve CD8 | TRB | 21981 | 19099 | 20752 | 20166 |
| a1_nTreg | 29 | young | naïve Treg | TRB | 12793 | 5162 | 8186 | 7864 |
| a3_nTreg | 32 | young | naïve Treg | TRB | 13118 | 5985 | 8970 | 8455 |
| a5_nTreg | 25 | young | naïve Treg | TRB | 37392 | 9688 | 19452 | 18304 |
| a7_nTreg | 26 | young | naïve Treg | TRB | 8616 | 5040 | 6711 | 6402 |
| a9_nTreg | 31 | young | naïve Treg | TRB | 18835 | 4155 | 8774 | 8122 |
| a10_nTreg | 26 | young | naïve Treg | TRB | 6115 | 3702 | 4862 | 4636 |
| a11_nTreg | 24 | young | naïve Treg | TRB | 5953 | 3713 | 4796 | 4585 |
| a14_nTreg | 30 | young | naïve Treg | TRB | 13530 | 7775 | 10302 | 9995 |
| a16_nTreg | 28 | young | naïve Treg | TRB | 68627 | 31224 | 46520 | 45241 |
| o1_nCD4 | 55 | old | naïve CD4 | TRB | 161318 | 59240 | 98618 | 93675 |
| o3_nCD4 | 83 | old | naïve CD4 | TRB | 203771 | 35233 | 85870 | 81362 |
| o5_nCD4 | 78 | old | naïve CD4 | TRB | 37447 | 9580 | 19077 | 17835 |
| o6_nCD4 | 50 | old | naïve CD4 | TRB | 218088 | 62896 | 118217 | 111009 |
| o1_nCD8 | 55 | old | naïve CD8 | TRB | 49604 | 25252 | 35822 | 33909 |
| o2_nCD8 | 75 | old | naïve CD8 | TRB | 8325 | 6027 | 7077 | 6818 |
| o3_nCD8 | 83 | old | naïve CD8 | TRB | 9313 | 3493 | 5635 | 5332 |
| o4_nCD8 | 78 | old | naïve CD8 | TRB | 12971 | 2274 | 5273 | 4931 |
| o6_nCD8 | 50 | old | naïve CD8 | TRB | 31003 | 18578 | 24504 | 22901 |
| o7_nCD8 | 75 | old | naïve CD8 | TRB | 6522 | 5515 | 5972 | 5823 |
| o1_nTreg | 55 | old | naïve Treg | TRB | 18300 | 4019 | 8681 | 8038 |
| o3_nTreg | 83 | old | naïve Treg | TRB | 19894 | 4773 | 9620 | 9150 |
| o5_nTreg | 78 | old | naïve Treg | TRB | 25664 | 5688 | 11910 | 10985 |
| o6_nTreg | 50 | old | naïve Treg | TRB | 26586 | 19074 | 22765 | 22236 |

## **Supplementary Figures**


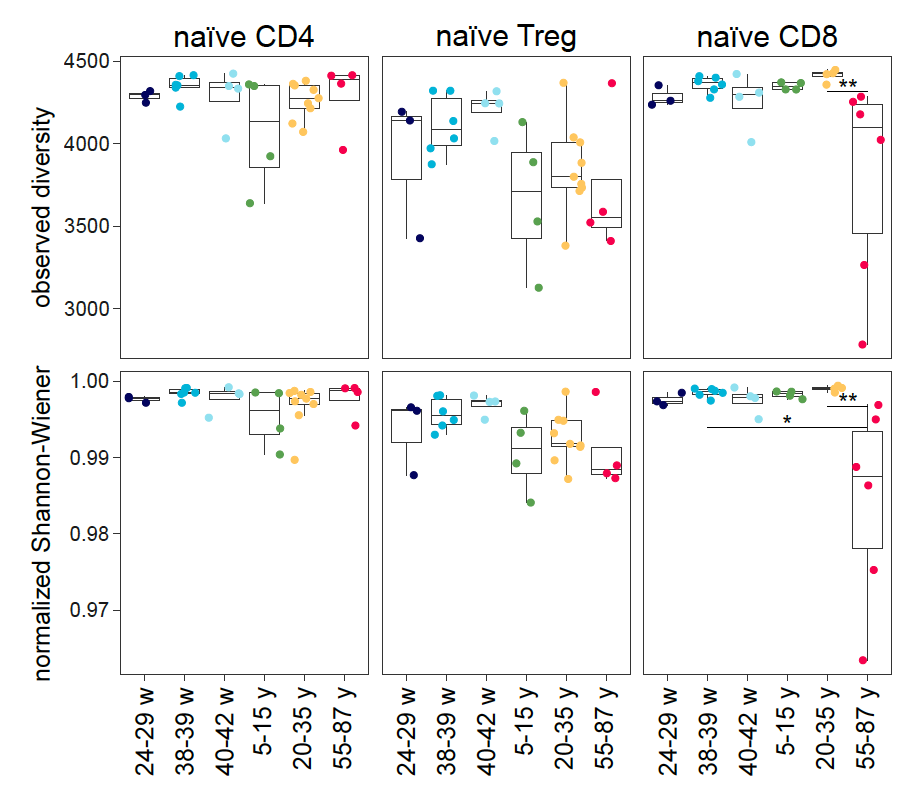


**Supplementary Figure 1.** Diversity and clonality (normalized Shannon-Wiener index, higher values mean less clonal) of TCRβ repertoires. Both metrics were calculated with downsampling to 4500 UMIs. Statistical significance was assessed using the Kruskal–Wallis test. Significance was calculated using the Kruskal-Wallis test with subsequent FDR correction and post hoc Dunn test. Only p-values < 0.05 are shown. ‘*’ – p ≤ 0.05, ‘**’ – p ≤ 0.01, ‘***’ – p ≤ 0.001, ‘****’ – p ≤ 0.0001.

**Supplementary Figure 2.** Boxplots show the frequency of individual amino acid residues within the five central positions of the CDR3β region in naïve CD4+ T cells across age groups. Analyses were performed separately for the repertoire of naïve CD8+ T cells in preterm (25–29 w.g.), term (38–39 w.g.), late (40–42 w.g.) UCB samples and PB from children (5–15 y.o.), adults (20–35 y.o.), and older adults (55–87 y.o.). Each panel corresponds to a single amino acid (single-letter code). Points represent individual donors. Statistical significance was assessed using the Kruskal–Wallis test. Significance was calculated using the Kruskal-Wallis test with subsequent FDR correction and post hoc Dunn test. Only p-values < 0.05 are shown. ‘*’ – p ≤ 0.05, ‘**’ – p ≤ 0.01, ‘***’ – p ≤ 0.001, ‘****’ – p ≤ 0.0001.

**Supplementary Figure 3.** Boxplots show the frequency of individual amino acid residues within the five central positions of the CDR3β region in naïve T_reg_ cells across age groups. Analyses were performed separately for repertoire of naïve CD8+ T cells in preterm (25–29 w), term (38–39 w), late (40–42 w) UCB samples and PB from children (5–15 y), adults (20–35 y), and older adults (55–87 y). Each panel corresponds to a single amino acid (single-letter code). Points represent individual donors. Statistical significance was assessed using the Kruskal–Wallis test. Significance was calculated using the Kruskal-Wallis test with subsequent FDR correction and post-hoc Dunn test. Only P-values  < 0.05 are shown. ‘*’ – P ≤ 0.05, ‘**’ – P ≤ 0.01.

**Supplementary Figure 4.** Boxplots show the frequency of individual amino acid residues within the five central positions of the CDR3β region in CD8^+^ T cells across age groups. Analyses were performed separately for the repertoire of naïve CD8+ T cells in preterm (25–29 w.g.), term (38–39 w.g.), late (40–42 w.g.) UCB samples and PB from children (5–15 y.o.), adults (20–35 y.o.), and older adults (55–87 y.o.). Each panel corresponds to a single amino acid (single-letter code). Points represent individual donors. Statistical significance was assessed using the Kruskal–Wallis test. Significance was calculated using the Kruskal-Wallis test with subsequent FDR correction and post hoc Dunn test. Only p-values < 0.05 are shown. ‘*’ – p ≤ 0.05, ‘**’ – p ≤ 0.01, ‘***’ – p ≤ 0.001, ‘****’ – p ≤ 0.0001.

**
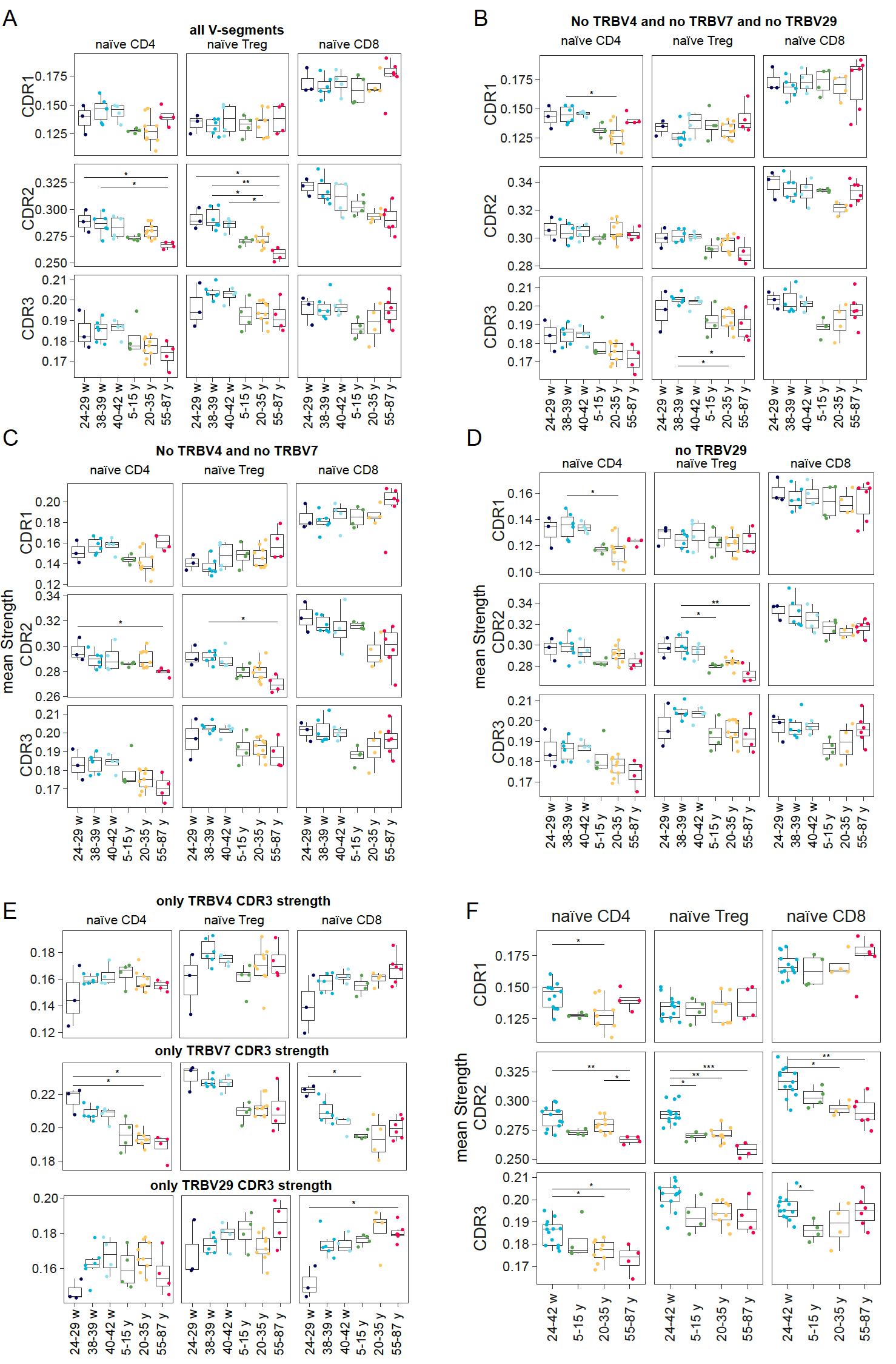
**

**Supplementary Figure 5.** **Analysis of predicted CDR binding strength in repertoires across age groups**. The average predicted binding strength of CDR1, CDR2 regions was calculated with normalization by amino acid length. Mean strength for CDR3 was calculated for five central amino acids in repertoires of naïve CD4⁺, naïve Treg, and naïve CD8⁺ T cells. Analyses were performed for the full functional repertoire: without depletion (A), after depletion of clonotypes carrying TRBV4, TRBV7, and TRBV29 (B), after depletion of clonotypes carrying TRBV4 and TRBV7 (C), and after depletion of clonotypes carrying TRBV29 (D). (E) Average CDR3 binding strength calculated for five central amino acid residues of TRBV4, TRBV7 and TRBV29 clonotypes only in repertoires of naïve CD4⁺, naïve Treg, and naïve CD8⁺ T cells. (F) Average CDR3 binding strength calculated for five central amino acid residues for merged UCB repertoires and other age groups. Significance was calculated using the Kruskal-Wallis test with subsequent FDR correction and post hoc Dunn test. Only p-values < 0.05 are shown. ‘*’ – p ≤ 0.05, ‘**’ – p ≤ 0.01, ‘***’ – p ≤ 0.001, ‘****’ – p ≤ 0.0001.

**Supplementary Figure 6. Distribution of CDR3β length in antigen-annotated TCR clonotypes from VDJdb.**

Histograms show the distribution of CDR3β amino acid length for TCR clonotypes annotated as specific to human autoantigens, CMV, EBV, HIV-1, and SARS-CoV-2. Clonotypes were extracted from VDJdb using a confidence score ≥ 1. In total, 872 CMV-, 546 EBV-, 808 HIV-, and 329 SARS-CoV-2-specific clonotypes were analyzed, together with 419 autoantigen-specific TCRs. The x-axis indicates CDR3β length (amino acids), and the y-axis represents normalized frequency.

**References.**

1. Shelyakin PV, Lupyr KR, Egorov ES, Kofiadi IA, Staroverov DB, Kasatskaya SA, et al. Naïve Regulatory T Cell Subset Is Altered in X-Linked Agammaglobulinemia. Front Immunol. 2021 Aug 19;12:697307.

2. Egorov ES, Kasatskaya SA, Zubov VN, Izraelson M, Nakonechnaya TO, Staroverov DB, et al. The Changing Landscape of Naive T Cell Receptor Repertoire With Human Aging. Front Immunol. 2018 Jul 24;9:1618.
